# Using the EMFIT Sensor in Geophysical Monitoring

**DOI:** 10.3390/s25216746

**Published:** 2025-11-04

**Authors:** Victorin-Emilian Toader, Constantin Ionescu, Iren-Adelina Moldovan, Alexandru Marmureanu

**Affiliations:** National Institute for Earth Physics, Călugăreni 12, 077125 Măgurele, Romania; viorel@infp.ro (C.I.); iren@infp.ro (I.-A.M.); marmura@infp.ro (A.M.)

**Keywords:** geophysical monitoring, acoustic emission monitoring, ferroelectret, PVDF

## Abstract

EMFIT, also referred to as EMFi, is a ferroelectret film related to polyvinylidene fluoride (PVDF) sensors. It is an electroactive polymer (EAP) based on a polyolefin structure and consists of three layers of polyester film. Its application in geophysical monitoring has not been reported in the literature. At present, EMFIT is mainly employed in ballistocardiography and medical sleep monitoring, as developed by the manufacturer Emfit Ltd. (Vaajakoski, Finland). Within the multidisciplinary monitoring network of the National Institute for Earth Physics (NIEP), EMFIT is used as a pressure sensor in combination with infrasound transducers and microphones deployed in seismic areas. The primary aim of this study is to evaluate its suitability for detecting seismic noise that precedes earthquakes, generated by rock fracturing associated with crustal deformation. Although similar studies have been reported, they have not involved the use of EMFIT sensors. The novelty of this approach lies in the large surface area and mechanical flexibility of the material. Beyond seismic forecasting, the research also examines whether this type of sensor can contribute to seismic monitoring as a complement to conventional instruments such as accelerometers, seismometers, and microbarometers. Data analysis relies primarily on spectral time-series methods and incorporates measurements from other acoustic sensors (microphones and microbarometers) as well as a weather station. The working hypothesis is the potential correlation between the recorded data and the presence of enhanced noise prior to the detection of seismic waves by standard seismic sensors. The target area for this investigation is Vrancea, specifically the Vrâncioaia seismic station, where multidisciplinary monitoring includes infrasound, radon, thoron, soil temperature, and atmospheric electrical discharges. Preliminary tests suggest that the EMFIT sensor may function as a highly sensitive device, effectively serving as an “ear” for detecting ground noise.

## 1. Introduction

This study investigates the performance and potential of sensors produced by Emfit Ltd. (Vaajakoski, Finland) for applications in geophysical monitoring, with particular emphasis on seismic forecasting. Experimental tests examined the frequency response of the sensors, their sensitivity to environmental parameters (temperature, humidity, and atmospheric pressure), transfer function, and their comparative performance against established instruments, including infrasound sensors, seismometers, accelerometers, geophones, and pressure microphones. The overarching objective is to strengthen the network of seismic precursors deployed at the Vrâncioaia seismic station in the Vrancea region.

To date, no published studies have reported on the use of this type of sensor for such applications. The National Institute for Earth Physics (NIEP) maintains a multidisciplinary database containing measurements of the magnetic field, telluric currents, borehole and soil temperature, infrasound, CO_2_, radon, ULF–VLF radio waves, and ground inclination, among others, collected through research projects exploring the feasibility of detecting earthquakes with magnitude Mw > 4 prior to their occurrence. Archived recordings acquired with EMFIT sensors, although unpublished, were also identified in the database. Re-examination of these data provided the impetus to resume this line of research, particularly in light of recent advances in digitalization and data processing.

Electromechanical film (EMFi) was invented in Finland in 1987 and represents the first cellular polymer electret film developed for commercial applications [1]. At present, EMFi or version EMFIT sensors are widely used in medical, surveillance, and healthcare fields, where they detect movement, activity, or physiological parameters such as blood pressure, pulse, respiration, vocal cord vibrations, and cardiac activity, as well as in smart bed systems for health and sleep monitoring [2,3,4,5].

The piezoelectric structure of EMFIT enables its dual use as a receiver and transmitter. It has been employed as a guitar microphone, as an actuator in audio systems, and in active noise cancellation (ANC) applications [2,6,7]. EMFIT has also been used as an ultrasonic transducer in radar systems [2,8], as a sonar head for autonomous robotic platforms [9,10], and in sports for measuring take-off force in ski jumping [11]. Another application involves smart seating, where sensors integrated into car seats enable contactless monitoring of vital signs, including capacitive electrocardiogram (cECG), ballistocardiography (BCG), and mechanical motion analysis [12].

The operating principle of EMFIT is based on thickness variations induced by external force or pressure, which generate electric charge and, consequently, a voltage across the electrodes [6,13]. Applications of acoustic and vibration-based electromechanical transducers have been reported across audio, ultrasonic, and infrasonic frequency ranges [14]. Furthermore, EMFIT has been integrated into active sound control (ACS) systems involving microphones and loudspeakers [7,15]. Since 1999, the VTT Technical Research Centre of Finland has developed and implemented EMFIT-based acoustic sensors and actuators in ACS and ANC applications, demonstrating effective noise reduction in buildings and transportation systems [16,17].

EMFIT sensors have also been applied in the automatic detection of lameness in cattle by analyzing pressure forces to differentiate between healthy and affected animals [18]. EMFi acoustic transducers developed by VTT have been investigated for airborne and underwater sound transmission, with superior results in air. It should be emphasized, however, that EMFi and EMFIT are not piezoelectric in the classical sense [19].

EMFIT belongs to the broader class of ferroelectrets, a term introduced by Bauer et al. [20] to describe materials combining the properties of ferroelectrics and space-charge electrets. This distinction is emphasized by Kärki and Lekkala [1] in their review of piezoelectric materials based on polyvinylidene fluoride (PVDF). They differentiate PVDF from cellular polymers such as EMFi, which mimic piezoelectricity through their electromechanical properties. The same authors conducted a comparative study of PVDF and EMFi sensor materials, highlighting their structural differences and distinct piezoelectric effects [21]. A concise review of ferroelectret materials is presented in [18], though EMFIT itself is not specifically mentioned. These materials have been utilized in air-coupled ultrasound transducers (ACUS) [22] and airborne ultrasonic arrays [23]. In robotics, ferroelectrets are particularly valued for tactile texture detection due to their thinness and mechanical flexibility [24]. The differences in sound quality between electret film (EMFIT) and piezoelectric guitar pickups are described by Miikka Tikander and Henri Penttinen in [25].

The similarity of EMFIT to piezoelectric sensors suggests its potential as a replacement for traditional geophones, with applications in detecting underground mining activity [26]. Related studies have examined the analysis of surface seismic waves (Rayleigh and Love) and the detection of anthropogenic emissions in the ELF band using piezoelectric films. Our research focuses on seismic noise generated by rock fracturing due to tectonic stress. EMFIT sensors could also be applied to landslide monitoring or the study of mud volcanoes in the Buzău region, in combination with CO_2_ and radon measurements, which are already being performed. Recent work by Ming Li et al. [27] highlights advances in flexible PVDF-based piezoelectric sensors for applications in health, motion tracking, sports monitoring, human–machine interaction (HMI), smart homes, and vibration, though seismic applications are not addressed. PVDF films have also been employed for surface crack measurements using Rayleigh wave analysis in the frequency domain [28]. The importance of Rayleigh waves is further emphasized by Qingling Du et al. [29], who investigated their time–frequency characteristics in simulated and real seismic noise signals—an area directly relevant to our study. The potential to record Rayleigh waves with EMFIT sensors opens perspectives for tomography based on ambient noise and estimated Green’s functions (EGF) [30,31]. Ambient seismic noise, together with surface waves, plays a critical role in probing Earth’s interior, [32,33,34,35] even in urban environments [36]. The applicability of EMFIT sensors in this context, however, requires further validation. During initial tests, several teleseisms generated by Mw > 6 earthquakes at epicentral distances greater than 7000 km were recorded; however, this evidence remains insufficient.

Cazzulani et al. [37] investigated the feasibility of replacing acoustic emission (AE) sensors with piezoelectric PZT patches as a cost-effective alternative for monitoring crack propagation in concrete. Such research broadens the range of potential applications for EMFIT sensors in acoustic emission studies.

The NIEP monitoring network is multidisciplinary and includes, in addition to seismic and geophysical monitoring, the monitoring of building structures (such as road crossings, residential buildings, and dams). Structural Health Monitoring (SHM) is another area where EMFIT sensors can be used alongside existing equipment (accelerometers, gyro inclinometers, microphones, displacement sensors, geoelectric radars, GNSS, InSAR, Lidar) to determine the dynamic characteristics of buildings (natural frequency, damping) and to track the evolution of these parameters over time. The monitoring covers both natural (earthquakes, landslides) and anthropogenic phenomena, aiming to mitigate risks and protect the population in affected areas. The use of EMFIT sensors in this field is in its early stages and currently focuses on recording the noise produced by building structures during earthquakes. Seismic sensors only detect seismic waves and are limited to microphones with a sampling rate of 25 kHz to 50 kHz, which are useful for recording the ‘scream’ of concrete and beam reinforcement. In this study, we used Bode diagrams to determine the transfer function based on an explosion; any modification indicates a change in the building structure. The use of ferroelectret sensors with piezoelectric properties is mentioned in the specialized literature. Pengcheng Jiao et al. present in [38] the use of the piezoelectric effect in sensing methods to detect structural responses. In the article [39], a flexible piezo-ferroelectret sensor for structural health monitoring (SHM) is designed and fabricated based on the piezoelectric response of an electret film. It can be replaced with an EMFIT, which has similar properties, even though it is not truly piezoelectric. Tests on cement mortar indicate a linear relationship between the damage index and crack widths. In this case, the focus is on the occurrence of cracks and the noise they produce in rocks as a result of soil deformation. Pedro M. Ferreira et al. [40] analyzed the “design, fabrication, construction and implementation of embedded sensors (ES)”. The aim is to monitor structures and structural components in real time and throughout their life cycle. In this application, they mention piezoelectric sensors (PS). The EMFIT could be embedded in concrete, respecting the linearity condition (5–60 N/cm^2^). Ye Hai Li et al. [41] are investigating the use of piezoelectric sensors in a monitoring network for monitoring the condition of multi-scale structures. EMFIT ferroelectrics can also be used in this context because they are flexible and can be customized in shape depending on the application. The main problem in the articles on SHM is that only the building is monitored, not the coupling with the ground. And the area where the foundation was built changes over time (settlement, the quality of the supporting surface is affected by water penetration, etc.).

A recent NIEP project aimed to locate explosions using microphones, which can be replaced with EMFIT sensors.

In summary, EMFIT sensors demonstrate versatility across a wide spectrum of applications, including seismic monitoring, although they are rarely mentioned in the existing literature. Our experimental tests, which included comparisons with acceleration, velocity, and infrasound sensors, provide insight into their response characteristics. The results suggest that EMFIT sensors hold promise for seismic forecasting and environmental noise analysis, complementing traditional instrumentation.

## 2. EMFIT Transducer and Experimental Tests

The EMFIT sensor is manufactured by Emfit Ltd. (Vaajakoski, Finland) in three configurations: L, R, and S. For research purposes, the company also provides EMFIT ferroelectret film, sometimes combined with ferroelectric films such as PVDF. In certain cases, sensor elements produced by Emfit Ltd. are incorporated into custom devices [42].

The EMFIT S-series sensor is a fully shielded device consisting of two layers of polyester film with screen-printed silver-paste electrodes. It is produced in both rectangular (10 × 20 mm) and circular (22 mm diameter) geometries (Emfit Ltd., S-series product page, https://www.sensors.emfit.com/copy-of-r-series-sensor accessed on 27 September 2025). The R-series sensors are fully shielded, low-mass, ribbon-type devices made from EMFIT ferroelectret film with three electrode layers on polyester films. Their standard dimensions are 19 mm in width, up to 6 m in length, and 0.4 mm in thickness (Emfit Ltd., R-series product page, https://www.sensors.emfit.com/copy-of-l-series accessed on 27 September 2025). The L-series sensors are fully shielded, thin, and large-area devices constructed from EMFIT ferroelectret film with three layers of polyester film and etched aluminum electrodes. Standard widths are 290 mm and 580 mm (Emfit Ltd., L-series product page, https://www.sensors.emfit.com/copy-of-publications-1 accessed on 27 September 2025).

EMFIT is an electroactive polymer (EAP) based on a polyolefin material manufactured through a continuous biaxial orientation process and expanded in thickness using a high-pressure gas diffusion–expansion (GDE) process (Emfit Ltd., printable EMFIT product page, https://www.sensors.emfit.com/copy-of-s-series-sensor accessed on 27 September 2025), [43]. This ferroelectret film represents the base element from which all sensor versions are constructed.

**Figure 1:**
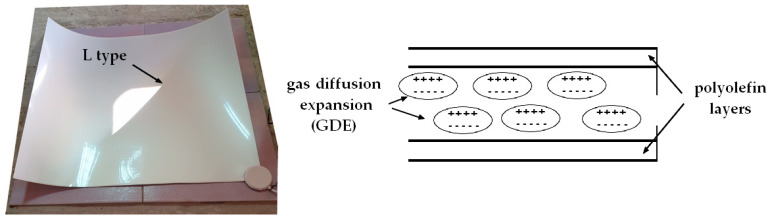
Emfit ferroelectret sensor, 3 layers of polyester film, L-series, simplified one air gap model (description https://www.sensors.emfit.com/copy-of-s-series-sensor accessed on 27 September 2025), microscopic image in [7].

The present study employed the L-series sensor, as illustrated in Figure 1.

Changes in the thickness of the EMFIT sensor generate a voltage across the electrodes, as the device responds specifically to thickness variations rather than bulk deformation. Functionally, it can be regarded as an active capacitor exhibiting piezoelectric-like behavior [1].

The fabrication process of this type of sensor has been described in detail by the VTT Technical Research Centre of Finland [7,13,16,17,44]. A schematic representation of the fabrication steps of a ferroelectret tactile sensor is provided in [14,45,46,47,48,49,50]. Hamdi et al. [14] offer a comprehensive explanation of the origin and manufacturing procedures of piezoelectric cellular polymer films, including the development of the cellular structure, foaming stages, saturation, nucleation, expansion, and stabilization.

Further contributions are noted in the broader literature. Bain and Chand, in the Transducers section, describe the EMFi film and ferroelectret sensors in general. More recently, Shi and Wagih [46] reported the first implementation of a wearable textile-based tactile sensor using polypropylene (PP) ferroelectret material for gesture recognition. In this work, PP film sheets produced by EMFIT Ltd. were integrated into gloves to enable contactless gesture detection.

### 2.1. Transducer Characteristics

The general characteristics of EMFIT sensors are presented in Table 1 the L-series used in tests.

Changes in the thickness determine the output voltage ΔV of an EMFIT sensor:

ΔV = (1/C) × S_q_ × ΔF (1)

where C is the capacitance, Sq the sensitivity of the sensor and ΔF the applied force [51].

S_q_ = Δσ/Δp (2)

where ∆σ is the change in the charge density on the electrodes and ∆p = ∆F/Ad is the amplitude of the dynamic pressure within the area Ad where the force is acting [2].

Equations (1) and (2) are only valid when the sensor is operating within its linear region. Table 1 specifies the operating force range, P < 300 N/cm^2^, and the operating temperature from −20 to +70 °C. The document [52] shows that EMFIT sensors are linear at least between 5 and 60 N/cm^2^ (standard deviation was <5%). It is also stated that the linearity depends on the number of EMFIT layers and the adhesives used to assemble the sensors.

Generally, the ferroelectret sensors’ size, dimension, surface, and direction of excitation [53], are important in AE applications, which is an advantage for EMFIT. Rayleigh waves (RW) receiving sensitivities depend on sensor size [54].

The experiments presented in this study were performed using an EMFIT L-series device (60 × 60 cm) supplied by Emfit Ltd. (Vaajakoski, Finland). The system was delivered with an impedance adapter and a preamplifier, providing sensitivities of 53 pC/N, 19.08 pC/Pa, and 86.7 µV/Pa, together with an analog high-pass filter with a cutoff frequency of 0.007 Hz.

The Chaparral Model 64 S is the reference equipment. The verification of EMFIT sensors in the infrasound spectrum is being pursued. Its technical specifications are in Table 2 (Operation manual for the Model 64 S Infrasound Sensor for use in the wide infrasound band, Chaparral Physics, Manual Revision 1, 28 April 2022).

**Figure 2:**
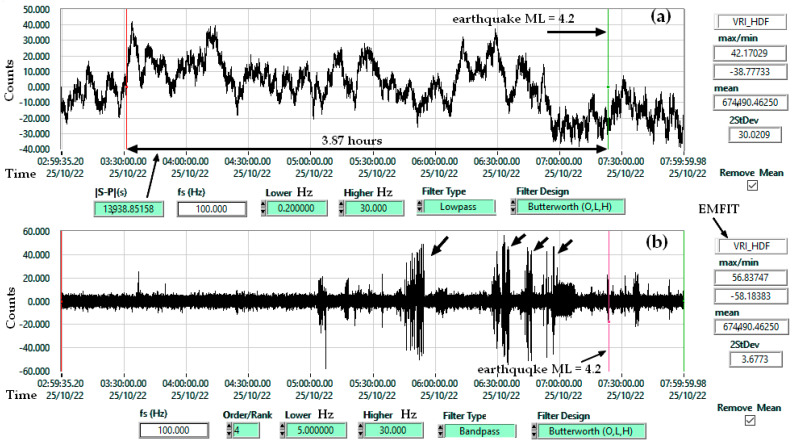
Emfit sensor (VRI_HDF) used in geophysical monitoring, earthquake ML 4.2, Latitude 45.8420, Longitude 26.6994, Depth 90.1 Km, 22 October 2025 07:23:26; (**a**) Lowpass Filter 0.2 Hz; (**b**) Bandpass Filter 5–30 Hz.

The primary objective of these tests was to evaluate whether EMFIT sensors can be employed for earthquake forecasting in conjunction with other dedicated monitoring instruments. Preliminary evidence from the NIEP archive supports this hypothesis. Figure 2 presents a representative recording obtained with this type of sensor in Vrâncioaia station.

In case (a), the first change in the amplitude of the time series filtered with a low-pass filter occurs almost 4 h before the earthquake. At low frequencies, a change in pressure on the sensor is more noticeable. In case (b), the focus is on highlighting the seismic noise specific to rock breakage. This noise is observed when testing construction materials at the moment of breakage under controlled pressure.

### 2.2. Experimental Tests

**Figure 3:**
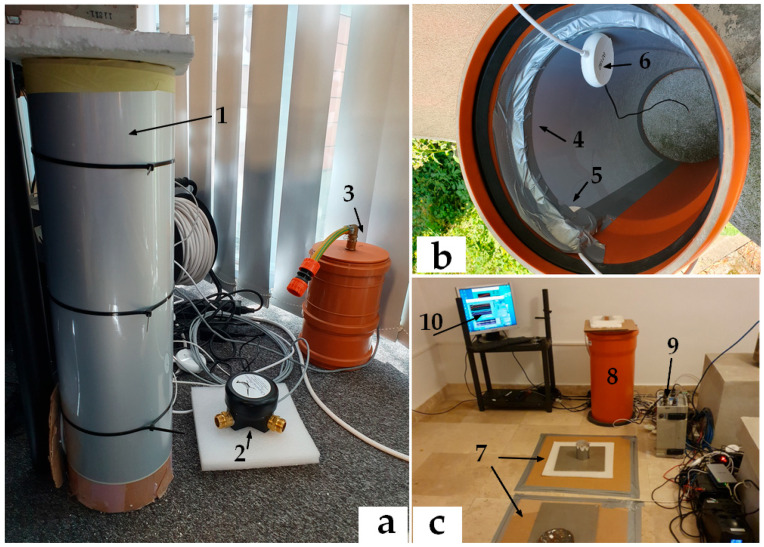
Test Emfit sensor: (**a**) (1) EMFIT sensor mounted externally on a vertical tube; (**a**) (2–3) infrasound equipment; (**b**) EMFIT sensor mounted internally; (**c**) horizontal installation.

**Figure 4:**
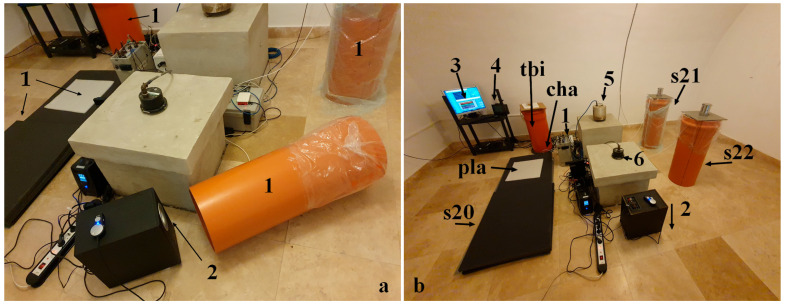
Experimental configuration; (**a**) EMFIT sensors mounted horizontally and vertically with an acoustic vibration generator; (**b**) test chamber configuration.

Comparative tests were conducted using infrasound sensors (Chaparral and LPM 5481–Druck), mounted in both horizontal and vertical orientations. Since the NIEP network includes infrasound arrays, it was essential to assess whether EMFIT sensors could adequately cover this frequency range. For reference, the same test room also housed a seismometer and an accelerometer. Figure 3 and Figure 4 illustrate the experimental setup and instrumentation.

The infrasound measurement tubes were constructed from PVC (polyvinyl chloride) with lengths ranging from 0.71 m to 1 m and an inner diameter of 0.315 m. Because the EMFIT sensor responds specifically to changes in its thickness, only one side of the film was exposed to ambient vibrations, while the opposite side was insulated. Mounting the sensor directly on a flat surface facilitates this isolation but requires more space. By contrast, the tube-based configuration is more compact, requiring only fixation with silicone. To minimize internal wave reflections, a sponge was inserted at the upper end of the tube, while the lower end was sealed, ensuring that the sensor recorded vibrations exclusively from below.

Numerous methods for sensor calibration are reported in the literature. Among these, the face-to-face transmitter–receiver method is described by Kanji Ono [55], along with reciprocity calibration methods, which are well established for acoustic transducers (hydrophones and ferroelectret devices). A related approach is the surface-to-surface method [56], which was employed in the present tests: a source transducer generated vibrations simultaneously recorded by a reference sensor and the EMFIT device under test. The reciprocity calibration method for acoustic emission transducers has been widely [55,57,58,59,60,61], although it is constrained by the Hill–Adams equation [55]. Rayleigh waves have also been used in acoustic emission calibration, as reported by Hatano [57] and others [54,58].

The experiments in this study focused on the frequency response of EMFIT sensors in the 5–45 Hz range. Similar comparative tests are described in the literature, including the characterization of EMFi microphones against reference B&K microphones [6], and multilayer EMFIT configurations (one, two, and three layers) [42]. Frequency response analysis using the surface-to-surface calibration method has also been presented by Prevorovsky and colleagues [56,62].

Figure 3 and Figure 4 illustrate the sensor configurations tested. In Figure 3a, the EMFIT sensor (1) was mounted on the exterior of a vertical tube, with Chaparral (2) and LPM 5481–Druck (3) sensors serving as references. Figure 3b shows the EMFIT sensor inside a vertical tube (4), connected to the impedance adapter (5) and preamplifier (6). In this case, vibrations were captured within the tube - a configuration not previously reported in the literature. Typically, EMFIT sensors are used in horizontal orientation, as shown in Figure 3c, (7). The same figure also depicts an EMFIT sensor mounted inside a tube (8) with a Chaparral transducer at its base, a 24 bits Kinemetrics K2 digitizer (9), and data acquisition software (10). The vibration source was provided by a software signal generator and an audio amplifier.

In Figure 4a, EMFIT sensors (1) were positioned both horizontally and vertically, with the audio speaker acting as the vibration source (2). The final configuration is shown in Figure 4b, where EMFIT sensors are marked according to the schematic (tbi = inner tube, cha = Chaparral, etc.). The vibration source (2) was oriented toward the floor, while environmental parameters (temperature, humidity, and pressure) were monitored by a weather station (4). An accelerometer and a seismometer, mounted on concrete pillars, are shown at positions (5) and (6), respectively. The ceiling of the test chamber has a semi-cylindrical shape with flat ends.

Because EMFIT sensors generate an electrical charge in response to changes in thickness, proper mounting is critical. Only one side of the sensor must be exposed to the vibration source, while the opposite side must remain insulated.

**Figure 5:**
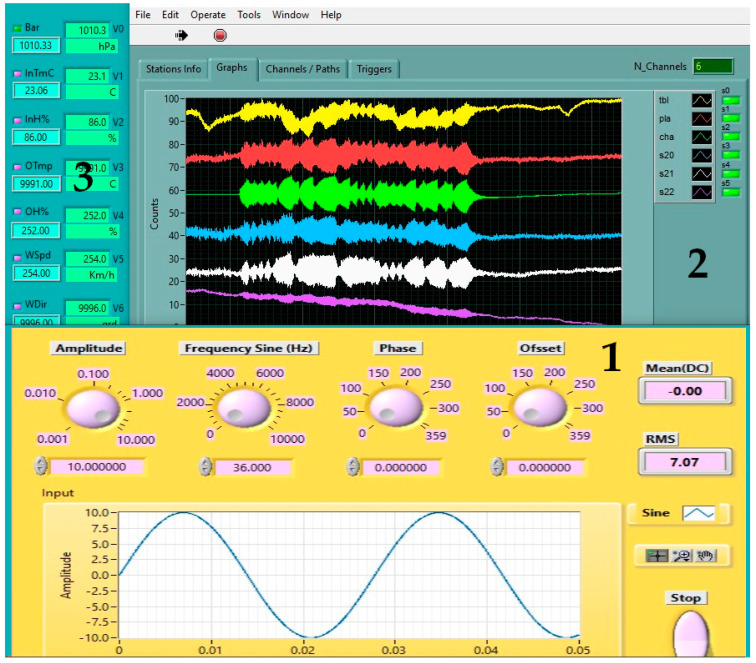
Experimental setup: (**1**) virtual signal generator; (**2**) data acquisition software; (**3**) meteorological monitoring station.

The signal generator was implemented in software (Figure 5(1)), providing test frequencies from 5 Hz to 45 Hz in 1 Hz increments. The preamplifier supplied with the EMFIT sensors has an upper cutoff frequency of 50 Hz, which, according to the Nyquist theorem, implies a sampling frequency of 100 Hz.

Data acquisition software (Figure 5(2)) recorded waveforms in SAC–BIN format. Environmental dependencies were monitored using a meteorological station (Figure 5(3)). A resonance peak at 36 Hz was observed under the test conditions.

## 3. Results

Tubes used for mounting EMFIT sensors exhibit resonant frequencies that may influence measurements. Depending on the design, the tubes may be open at both ends, open at only one end (Figure 4a), or completely closed (Figure 4b). The sensors can be mounted externally (Figure 3a) or internally (Figure 3b,c), provided that one side is insulated from external vibrations.

For a closed cylinder, the resonant frequency is given by the relationship ([63]):

(3) $$f=\frac{n\times v}{4\times (L+0.3\times d)}$$

where

f = resonant frequency, Hz;
n = harmonic 1, 3, 5 …;
v = speed of sound, 340 m/s;
L = tube length, m;
d = tube diameter, m;

0.4 X d represents the end correction of a closed tube or pipe.

For L = 0.71 m and d = 0.315 m (as in Figure 3 and Figure 4), the first resonant frequency is 105.65 Hz, outside the test range. For the maximum tube length of 1 m, the first resonance is 77.66 Hz, still above the frequencies tested.

**Figure 6:**
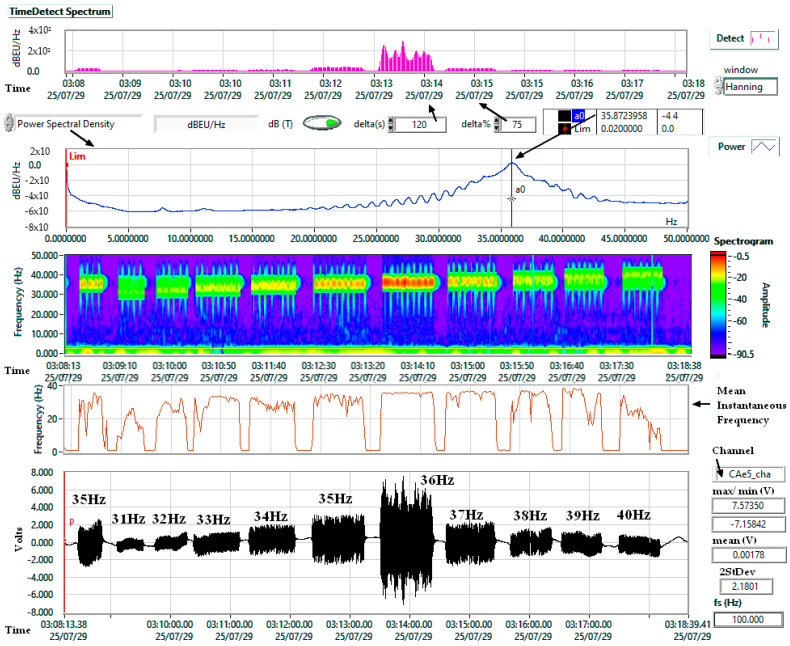
Test signals, frequency response of the Chaparral (cha) sensor, example for the range 31–40 Hz.

Figure 6 illustrates an example of a frequency response test for the 31–40 Hz range, although the experiments were performed from 5 to 45 Hz with 1 Hz increments. A clear maximum at 36 Hz is visible in both the Power Spectral Density (PSD) and the Short-Time Fourier Transform (STFT) spectrogram.

**Figure 7:**
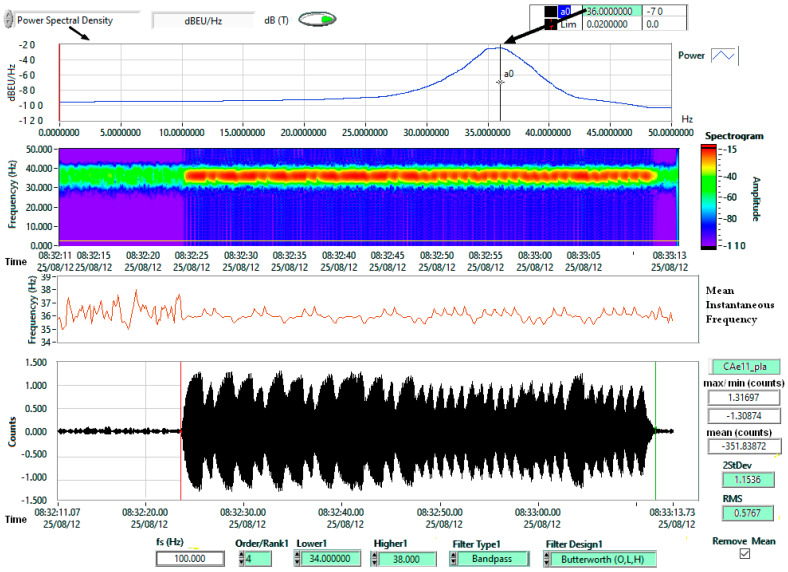
Test signal, frequency response of the Emfit sensor ‘pla’ (Figure 4b) for 36 Hz.

Each frequency was analyzed using a ±2 Hz Butterworth band-pass filter (Figure 7).

RMS values and double the standard deviation (2σ or 2StDev), a parameter widely adopted as a threshold indicator [64,65], were calculated for each sensor shown in Figure 4b. The results are summarized in Table 3.

Standard deviation (σ) is critical for estimating measurement standard uncertainty [66,67,68]. Within ±2σ, there is approximately a 95.4% probability that the true value lies within this range (https://itu.physics.uiowa.edu/courses/reporting-uncertainties accessed on 27 September 2025). Twice the standard deviation (±2σ) is applied in many cases, like a trigger limit [69,70,71,72].

**Figure 8:**
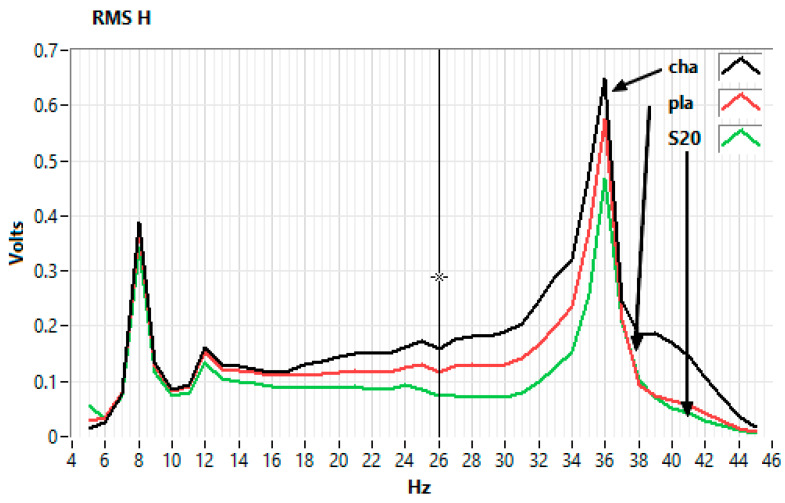
Frequency response of the horizontally mounted Emfit sensors (pla, s20) and the Chaparral reference (cha).

**Figure 9:**
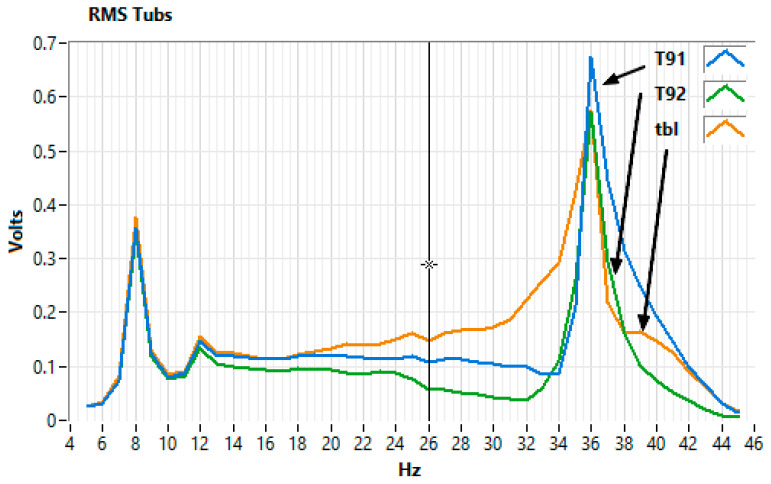
Frequency response of Emfit sensors mounted vertically in tubes (tbi, s21, s22).

Figure 8 and Figure 9 provide a visual representation of the results. Figure 8 shows the frequency response of horizontally mounted EMFIT sensors (pla and s20, Figure 4b) in comparison with the Chaparral reference sensor (cha, Figure 3c and Figure 4b).

Figure 9 presents the response of EMFIT sensors mounted vertically inside tubes (s21 and s22, Figure 4b). In both cases, the response curves closely resemble those of the Chaparral reference.

**Figure 10:**
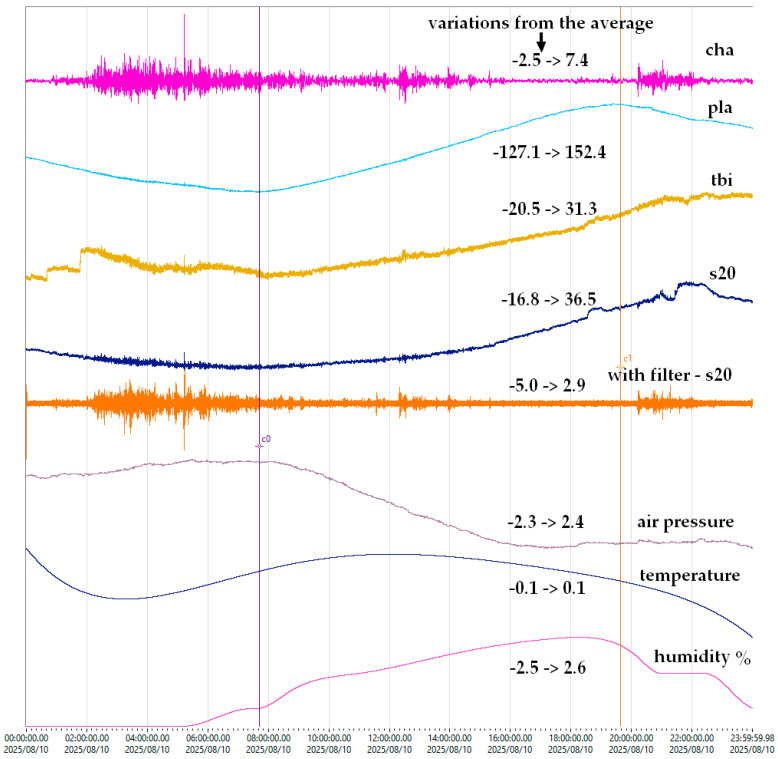
Dependence of Emfit sensors on environmental conditions.

Environmental dependencies were monitored with a meteorological station (Figure 4b(4)). Figure 10 shows daily variations in EMFIT and Chaparral sensor output.

**Figure 11:**
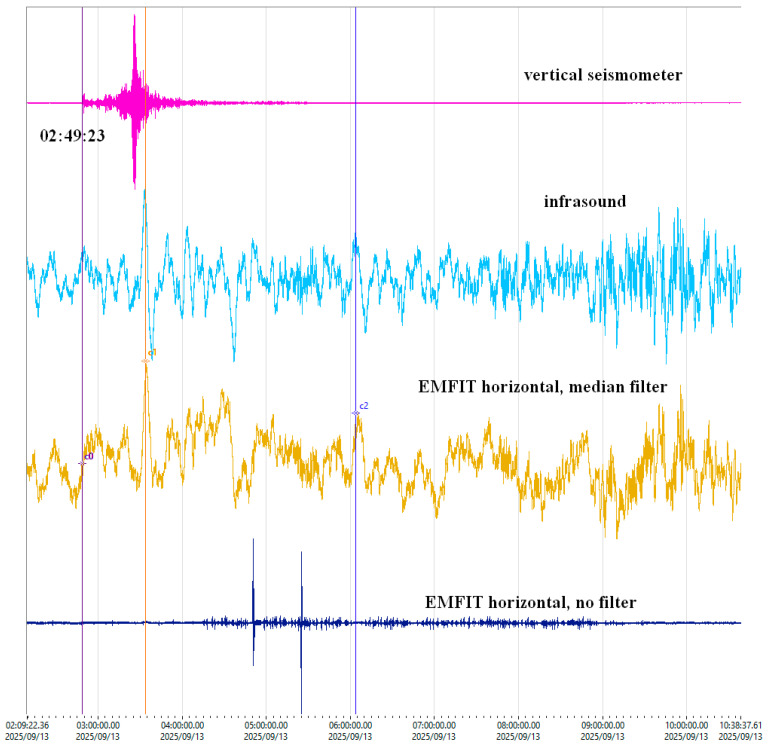
Recording of Rayleigh waves produced by an earthquake located in Kamchatka by an infrasound sensor and an Emfit.

The filtered signal “s20” demonstrates a clear correlation with the Chaparral sensor, confirming environmental influence. Further, Figure 11 demonstrates the recording of Rayleigh waves from a distant earthquake in Kamchatka (epicentral distance > 7000 km), comparing seismometer, infrasound, and EMFIT signals.

The unfiltered EMFIT signal in Figure 11 shows that it has a larger bandwidth than the Chaparral infrasound. After removing the local noise by filtering, a waveform similar to a maximum on the S of the seismic wave results, but the amplitudes are still small.

**Figure 12:**
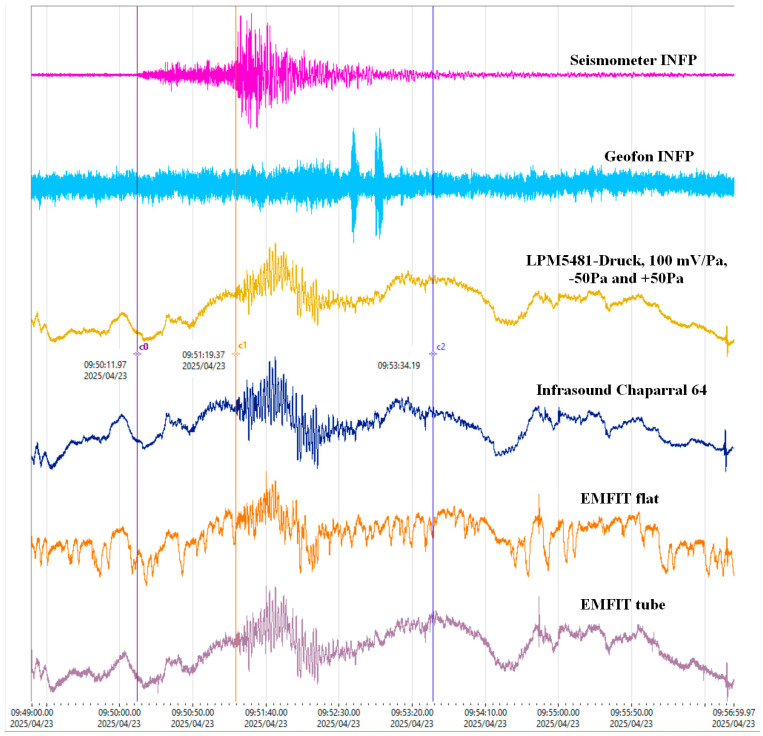
Recording of a 6.2 Mw earthquake located in the Sea of Marmara, 44 km SW of Esenyurt, Turkey, on 23 April 2025, at 09:49 UTC, with infrasound and Emfit sensors.

Figure 12 presents a 6.2 Mw earthquake in the Sea of Marmara (23 April 2025, 09:49 UTC), recorded by seismometers, geophones, infrasound sensors, and EMFIT sensors mounted both in tubes and flat on the ground (Figure 3a).

The example in Figure 12 refers to an earthquake with the epicenter located approximately 480 km from the location where the recording was made. It demonstrates that the best results can be obtained with EMFIT sensors if the vibrations reach their surface and produce a change in their thickness. In the case of Figure 12, this was achieved with an object that oscillated on the surface of the film and a tube that vibrated. The geophone response has a small amplitude, even though it was placed on the EMFIT sensor flat.

**Figure 13:**
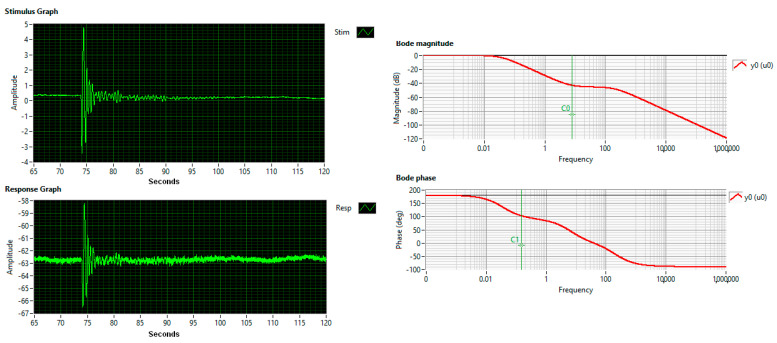
Bode Plots, Stimulus explosion (‘cha’), Response EMFIT ‘pla’.

JIRI KEPRT and PETR BENES compare the main methods for calibrating acoustic emission (AE) sensors in this article [73]. A calibration method is standardized by ASTM E 178 and ASTM E 976 [74] and requires a reference sensor with known characteristics. Calibration involves comparing the results from the reference transducer and the transducer under test. The transfer function, relation (4), and Bode plots are shown in Figure 13. The input signal is a shock produced by an explosion at a distance of 2 km, which is recorded by the Chaparral sensor (‘cha’, the reference) and the EMFIT sensors (‘pla’).

(4) $$y\left(s\right)=\frac{6.90969s-294.546}{0.00334077s2+4.35419s+1}\;u(s)$$

## 4. Discussion

The preceding section presented the equipment, test conditions, and results, with emphasis on frequency response, the potential use of EMFIT sensors in the infrasound domain, and their ability to capture Rayleigh waves relevant to seismic tomography.

The first consideration concerns the test environment. Experiments were carried out in a basement, where temperature fluctuations were minimal (Figure 10). The test location, at 44.410700° latitude and 26.093800° longitude, is in an area without seismic activity, faults, or geological anomalies. The ambient noise is composed of road traffic and the trees in the park where the building is located. The chamber was not acoustically isolated, allowing reflections from the semi-cylindrical ceiling and flat ends, which likely contributed to the resonant features in Figure 8 and Figure 9. These are not intrinsic to the Chaparral reference sensor but reflect the test setup. Ambient noise from outside the chamber was also present, but its effect was reduced by applying a ±2 Hz band-pass filter (Figure 7).

A second peculiarity relates to the audio test signal. As shown in Figure 6 (spectrogram) and Figure 7 (time series), the software signal generator did not produce a continuous wave but generated bursts at programmed frequencies. While different from laboratory-grade signal generators, the results remain valid because the Chaparral reference sensor was measured under identical conditions. Thus, the tests represent realistic working conditions rather than strictly controlled laboratory experiments, such as those described in [62].

Differences in sensor placement (Figure 4b) also contributed to variations. For example, the Chaparral sensor was mounted inside a tube, whereas EMFIT sensors had different positions relative to the source. This is reflected in Figure 8 and Figure 9, where deviations (e.g., at 39.5 Hz) appear consistently across both the Chaparral (cha) and EMFIT sensors (tbI).

Figure 10 highlights the sensitivity of EMFIT sensors to atmospheric pressure, consistent with their response to thickness variations under air pressure. In both Figure 10 and Figure 11, a high-pass Butterworth filter at 2 Hz was applied to remove the DC component of the EMFIT signal. The observed correlation with Chaparral confirms EMFIT’s capability in the infrasound domain. Under such conditions, the sampling rate could be reduced from 100 Hz to 10 Hz, enabling smaller file sizes, use of lower-cost digitizers, and reduced computational resources. For example, in Figure 2 a 16-bit acquisition module with a 1 Hz sampling rate was used. This would require the EMFIT preamplifier filter to be adjusted accordingly.

The ability to detect Rayleigh waves (Figure 11) is particularly significant, as it suggests that EMFIT sensors could be applied in seismic tomography. However, more data and additional sensors are needed to validate this.

The recording of the Marmara earthquake (Figure 12) was facilitated by specific conditions: one EMFIT sensor was mounted externally (Figure 3a), while another was coupled with an oscillating object, enhancing the signal.

Finally, the use of EMFIT sensors mounted inside or outside tubes represents a novel approach. The primary aim is to capture waves propagating through the ground. A buried tube with an externally mounted EMFIT sensor could enhance coupling with the soil. However, using closed tubes in outdoor environments may introduce issues such as condensation, unless proper thermal insulation is applied. EMFIT sensors could also be adapted for aquatic environments, provided the electronics are adequately protected. In this case, the water level itself could be monitored, with the caveat that wave direction must remain perpendicular to the sensor surface. Numerical simulations, such as those described in [53], further demonstrate how sensor dimensions and impact direction influence acoustic emission sensor performance—considerations equally relevant for EMFIT.

Comparative tests demonstrated that EMFIT sensors are capable of operating in the infrasound range and of recording Rayleigh waves generated by earthquakes, with responses comparable to those of the Chaparral reference sensor. Both tube-based and floor-mounted configurations produced similar response curves, with observed differences primarily attributable to chamber resonances and sensor positioning relative to the vibration source.

The analyses revealed a clear correlation between EMFIT signals and those of the reference sensor, including sensitivity to atmospheric pressure variations. The application of a 2 Hz high-pass filter successfully removed the continuous component, further confirming the suitability of EMFIT sensors for low-frequency monitoring. The recordings of Rayleigh waves from the Kamchatka earthquake and the Marmara Sea seismic event provide additional evidence of their applicability in seismological studies, with the added advantage of reduced sampling rates and lower data acquisition costs.

## 5. Conclusions

This study demonstrates that EMFIT sensors can be effectively employed for recording infrasound and Rayleigh-type seismic waves, yielding results comparable to established instruments such as the Chaparral and LPM 5481–Druck. The experimental setups—both on flat surfaces and in tubes—highlight the versatility of these sensors for diverse applications. One research direction we are currently pursuing is the use of EMFIT sensors in vertical tubes containing water. This approach is applied in geophysical measurements in both closed and open water wells, where geophone chains are inserted to measure velocity profiles as a function of depth.

The possibility of using EMFIT sensors for recording surface Rayleigh waves is interesting because it is applicable in noisy seismic tomography and in detecting subsurface activity. We chose long-range teleseismic to have mainly surface waves. Recordings with EMFIT sensors are much noisier than traditional ones. The specialized literature generally refers to piezoelectric film sensors (EMFIT is not a true piezoelectric sensor) and PVDF films, which also led to our tests. So, it is preferable to use broadband seismic sensors and use EMFITs if they are not available.

The tests were conducted using an infrasound sensor as a reference. To enable comparison and determine a transfer function, the same response area was selected, but differences remain, as shown in Figure 11 and Figure 12. In the NIEP network of infrasound sensors, the signal is captured from the air. The EMFIT sensor functions as an “ear” for detecting ground noise and is isolated from air vibrations by sound-absorbing materials. Old data from the NIEP database indicate that EMFIT sensors can be used, but the relevant research has been lost, if it ever existed. Comparing the old records with the new ones suggests that the sensors may not be of the same type, even if they share the same name.

The findings support the use of EMFIT sensors as a complementary and cost-effective alternative for geophysical monitoring. Nonetheless, further research and extended field testing are required to fully validate their large-scale applicability.

## Figures and Tables

**Table 1:** L-series sensor technical specifications (https://www.sensors.emfit.com/copy-of-publications-1, accessed on 27 September 2025).

Property	Symbol	Value	Unit	Tolerance	Conditions
Storage temperature	Ts	from −40 to +50	°C		
Operating temperature	Tr	from −20 to +70	°C		
Thickness	D	0.4	mm	±5%	
Sensitivity ^1^	Sq	25	pC/N	±20%	normal force
Relative permittivity	er	1.1		±10%	@ 10 kHz
Capacitance	C	22	pF/cm^2^	±5%	@ 1–150 kHz, EQV
Tensile strength at break, TD		535	N	±10%	ISO-527-1
Elongation at break, TD		20	%	±20%	
Young modulus, TD		1	MPa	±50%	
Operating force range	P	<300	N/cm^2^		
Standard width	W	290/580	mm	±1%	
Sample length ^2^	L	300	mm	±1%	

^1^ Loss of sensitivity is <20% after the following temperature cycles as follows: 11 h at −20 °C, 1 h at +20 °C, 11 h at +70 °C, 1 h at +20 °C, 28 cycles; and 1 h at −20 °C, 1 h at +70 °C, 10 cycles. ^2^ Manufactured in rolls, width 580 mm, divided into rectangular areas with a size of 290 × 300 mm, which are electrically connected. Can be cut to shorter sensors.

**Table 2:** Chaparral Model 64 S Specifications.

Nominal Sensitivity: Standard	0.08 Volts/Pa @ 1 Hz, 250 Pa Full Scale Range
Output: Output type	Differential
Maximum	20 volts peak-to-peak (signal+ to signal−), 10 volts max signal to ground. With no signal applied, the outputs idle at +5.5 V
Frequency Response Self-noise	Flat to within 3 dB from 0.01 Hz to 245 Hz. Less than 1.3 × 10^−7^ Pa^2^/Hz @ 1 Hz (−69 dB, relative to 1 Pa), Less than 1.7 mPa RMS 0.1 to 200 Hz, less than 0.5 mPa RMS 0.5 to 2 Hz
Dynamic range	110 dB @ 0.5 Hz to 2 Hz
Output Impedance	150 Ω non-reactive, (recommend load > 10 kΩ) (Recommended less than 1.0 µF capacitive loading).
Short circuit protected	Momentary shorts from signal+ to signal−, and signal to ground
Power Requirements: DC Source	12 volts (11.25–20 volts) DC, reverse voltage protected. Battery-powered operation is recommended to optimize noise performance.
Current Drain	Less than 15 mA @ 12 V (Less than 168 mW)
Physical	The sensor will function in any position or attitude. Sealed to IP-67 with acoustic inlets sealed and mating electrical connector or cap installed. Preferred orientation for proper venting: manifold
Operating Temperature	down −40 °C to +65 °C
Humidity	95% (non-condensing).
Dimensions	3.86″ diameter, 3.1″ tall (not including the Garden Hose Inlets) (9.8 cm diameter, 7.8 cm tall)
Weight	1.25 lbs (0.57 kg)
Std Acoustic inlets	3-port manifold, standard US Garden Hose Thread

**Table 3:** Frequency response of EMFIT sensors, filter used bandpass Butterworth +/− 2Hz relative to testing frequency. The locations of the sensors are shown in Figure 4.

Sensor	cha		pla		s20		s21		s22		tbI	
f(Hz)	RMS	2StDev	RMS	2StDev	RMS	2StDev	RMS	2StDev	RMS	2StDev	RMS	2StDev
5	0.0144	0.0287	0.0285	0.057	0.057	0.0474	0.0254	0.0508	0.0253	0.0507	0.0246	0.0493
6	0.0248	0.0496	0.0339	0.0678	0.0321	0.0642	0.0309	0.0618	0.0313	0.0626	0.0349	0.0697
7	0.0775	0.1551	0.0787	0.1573	0.0738	0.1475	0.0745	0.1491	0.0732	0.1465	0.0808	0.1617
8	0.3879	0.7759	0.3769	0.7539	0.3434	0.6868	0.3563	0.7126	0.349	0.6981	0.3762	0.7525
9	0.1335	0.2671	0.1289	0.2578	0.1169	0.2337	0.1237	0.2475	0.12	0.2399	0.1294	0.2588
10	0.0855	0.171	0.0832	0.1664	0.0747	0.1494	0.0806	0.1613	0.0766	0.1531	0.0842	0.1684
11	0.0934	0.1868	0.0895	0.179	0.0794	0.1589	0.0868	0.1736	0.0808	0.1617	0.0912	0.1825
12	0.1626	0.3252	0.1536	0.3072	0.1335	0.2671	0.1476	0.2952	0.1345	0.2689	0.1565	0.3129
13	0.1294	0.2589	0.1212	0.2424	0.1036	0.2072	0.1192	0.2385	0.1035	0.207	0.1245	0.249
14	0.1281	0.2563	0.1193	0.2387	0.0987	0.1975	0.1192	0.2385	0.0996	0.1993	0.1238	0.2475
15	0.1224	0.2449	0.1157	0.2314	0.0954	0.1908	0.117	0.234	0.0957	0.1913	0.1181	0.2363
16	0.1164	0.2329	0.1107	0.2215	0.0893	0.1786	0.1132	0.2265	0.0923	0.1847	0.113	0.2259
17	0.1193	0.2386	0.1093	0.2186	0.0866	0.1732	0.1139	0.2278	0.0901	0.1802	0.1131	0.2263
18	0.1299	0.2598	0.1118	0.2237	0.088	0.1761	0.1177	0.2354	0.095	0.19	0.1222	0.2445
19	0.136	0.2721	0.1141	0.2283	0.0877	0.1755	0.1209	0.2418	0.0946	0.1892	0.1277	0.2554
20	0.1445	0.2889	0.1176	0.2352	0.0879	0.1759	0.1177	0.2355	0.0937	0.1873	0.1344	0.2688
21	0.1501	0.3003	0.1198	0.2396	0.0898	0.1795	0.1189	0.2378	0.0889	0.1779	0.141	0.282
22	0.1492	0.2984	0.1166	0.2333	0.0843	0.1686	0.1157	0.2314	0.0857	0.1713	0.1393	0.2786
23	0.1496	0.2993	0.116	0.232	0.0852	0.1704	0.1129	0.2259	0.0906	0.1812	0.1408	0.2817
24	0.1611	0.3222	0.1234	0.2467	0.0939	0.1878	0.1147	0.2295	0.0886	0.1772	0.1515	0.3029
25	0.1742	0.3484	0.1292	0.2584	0.0850	0.1701	0.1198	0.2397	0.0773	0.1546	0.1618	0.3236
26	0.1584	0.3168	0.1170	0.2340	0.0727	0.1454	0.1076	0.2153	0.0558	0.1116	0.1480	0.2960
27	0.1753	0.3506	0.1263	0.2526	0.0741	0.1481	0.1134	0.2268	0.0567	0.1134	0.1623	0.3246
28	0.1819	0.3638	0.1292	0.2585	0.0713	0.1426	0.1141	0.2282	0.0516	0.1033	0.1672	0.3344
29	0.1817	0.3634	0.1283	0.2567	0.0700	0.1399	0.1087	0.2175	0.0483	0.0966	0.1674	0.3347
30	0.1894	0.3788	0.1311	0.2622	0.0714	0.1428	0.1038	0.2077	0.0411	0.0822	0.1726	0.3453
31	0.2032	0.4063	0.1407	0.2814	0.0785	0.1570	0.0984	0.1968	0.0399	0.0798	0.1865	0.3731
32	0.2475	0.4950	0.1668	0.3337	0.0999	0.1998	0.0995	0.1990	0.0375	0.0751	0.2229	0.4459
33	0.2881	0.5762	0.1985	0.3970	0.1246	0.2492	0.0842	0.1685	0.0588	0.1177	0.2585	0.5171
34	0.3197	0.6395	0.2345	0.4691	0.1519	0.3037	0.0837	0.1674	0.1103	0.2206	0.2913	0.5827
35	0.4783	0.9567	0.3733	0.7466	0.2588	0.5177	0.2131	0.4263	0.2596	0.5193	0.4254	0.8508
36	0.6502	1.3005	0.5767	1.1536	0.4670	0.9340	0.6738	1.3477	0.5723	1.1446	0.5758	1.1517
37	0.2426	0.4853	0.2128	0.4257	0.2072	0.4144	0.4438	0.8876	0.2949	0.5899	0.2169	0.4339
38	0.1829	0.3659	0.0926	0.1852	0.1028	0.2056	0.3145	0.6291	0.1622	0.3243	0.1615	0.3231
39	0.1870	0.3740	0.0734	0.1468	0.0718	0.1437	0.2488	0.4977	0.1014	0.2028	0.1642	0.3284
40	0.1688	0.3376	0.0655	0.1310	0.0518	0.1037	0.1934	0.3868	0.0735	0.1470	0.1470	0.2939
41	0.1447	0.2895	0.0565	0.1129	0.0411	0.0822	0.1444	0.2887	0.0517	0.1033	0.1255	0.2509
42	0.1064	0.2128	0.0422	0.0843	0.0277	0.0553	0.0997	0.1995	0.0357	0.0714	0.0920	0.1839
43	0.0710	0.1421	0.0290	0.0581	0.0195	0.0391	0.0639	0.1277	0.0196	0.0391	0.0611	0.1223
44	0.0359	0.0719	0.0151	0.0301	0.0106	0.0212	0.0302	0.0603	0.0091	0.0181	0.0308	0.0615
45	0.0180	0.0359	0.0081	0.0161	0.0058	0.0117	0.0147	0.0294	0.0053	0.0107	0.0158	0.0317

## Data Availability

The raw data supporting the conclusions of this article will be made available by the authors on request.
